# Fluorogenic Aptamer-Based Hybridization Chain Reaction for Signal-Amplified Imaging of Apurinic/Apyrimidinic Endonuclease 1 in Living Cells

**DOI:** 10.3390/bios14060274

**Published:** 2024-05-27

**Authors:** Meixi Liu, Yunjie Tan, Chen Zhou, Zhaoming Fu, Ru Huang, Jin Li, Le Li

**Affiliations:** 1 State Key Laboratory of Digital Medical Engineering, School of Biomedical Engineering, Hainan University, Sanya 572024, China; liumeixi@hainanu.edu.cn (M.L.); tanyunjie@hainanu.edu.cn (Y.T.); zhouchen2021@hainanu.edu.cn (C.Z.); fuzhaoming@hainanu.edu.cn (Z.F.); 2 Key Laboratory of Biomedical Engineering of Hainan Province, One Health Institute, Hainan University, Sanya 572024, China; 3 Department of Painology, Hainan Cancer Hospital, Haikou 570311, China; 4 NHC Key Laboratory of Tropical Disease Control, Hainan Medical University, Haikou 571199, China; leli@hainmc.edu.cn

**Keywords:** fluorescence imaging, hybridization chain reaction, signal amplification, APE1

## Abstract

A fluorogenic aptamer (FA)-based hybridization chain reaction (HCR) could provide a sensitive and label-free signal amplification method for imaging molecules in living cells. However, existing FA-HCR methods usually face some problems, such as a complicated design and significant background leakage, which greatly limit their application. Herein, we developed an FA-centered HCR (FAC-HCR) method based on a remote toehold-mediated strand displacement reaction. Compared to traditional HCRs mediated by four hairpin probes (HPs) and two HPs, the FAC-HCR displayed significantly decreased background leakage and improved sensitivity. Furthermore, the FAC-HCR was used to test a non-nucleic acid target, apurinic/apyrimidinic endonuclease 1 (APE1), an important BER-involved endonuclease. The fluorescence analysis results confirmed that FAC-HCR can reach a detection limit of 0.1174 U/mL. By using the two HPs for FAC-HCR with polyetherimide-based nanoparticles, the activity of APE1 in living cells can be imaged. In summary, this study could provide a new idea to design an FA-based HCR and improve the performance of HCRs in live cell imaging.

## 1. Introduction

Fluorogenic aptamer (FA) is an oligonucleotide that can specifically bind and activate unique fluorescent dyes. FA possesses many excellent properties, including high brightness, spectral tunability, and photobleaching resistance [1,2,3]. The first fluorogenic RNA aptamer was first selected in 2011 [3]. In the following decade, various FAs with different sequences and ligand molecules were selected, such as Spinach [3], Broccoli [4,5,6,7], Lettuce [8], Pepper [9], etc. These label-free fluorescence probes have been used for imaging targeting RNA’s transcription, localization, and translation [6,7]. Considering that RNA aptamers are susceptible to degradation by RNases, which makes them difficult to use, researchers selected a fluorogenic DNA aptamer named Lettuce as an alternative [8]. The analysis results showed that the DNA aptamer–DFHBI-1T complexes have dissociation constants (K_D_) ranging from 350 to 960 nM, which is comparable to the RNA aptamers. The quantum yields ranged from 0.045 to 0.105. Furthermore, the researchers analyzed the co-crystal structure of Lettuce bound to DFHBI-1T to show how DNA can form elaborate 3D structures without using RNA-like tertiary interactions [10]. In comparison with fluorescent proteins and dyes whose fluorescences are “always on” once excited, the sequence-dependent luminescence characteristics of FAs makes them more flexible for designing a “signal switch” for biosensing and imaging. Specifically, FAs can be divided into two nonfluorescent fragments that can become fluorescent after co-localization to a target [8,11,12]. This recognition mode can further reduce background leakage caused by the target-independent interaction between aptamers and fluorophores, thus improving the specificity.

A hybridization chain reaction (HCR) is an entropy-driven self-assembly system that can be used to amplify the detection signal [13,14,15,16]. Benefiting from high efficiency and simplicity, HCRs have been broadly applied for imaging molecules in living cells [17,18]. Most studies perform an HCR with two hairpin probes (HPs), wherein the HP has a dye and a quencher at its stem so that the dye can be quenched. Typically, one HP (HP1) is unfolded by targeting and then hybridizing it with the other HP (HP2), which can further unfold another HP1. As a result, nicked double-stranded HCR products with various lengths will be formed. In the HCR products, the dye on the HP is separated with the quencher, and thereby, its fluorescence is turned on [19]. Although effective, covalent modifications increase the cost and complexity of the probe preparation. More importantly, it is necessary to design and screen the location of the dye and the quencher to achieve an optimal signal-to-background ratio (S/B).

Additionally, FA can provide a label-free way to “turn-on” fluorescence imaging. For instance, in a target-recycled DNA circuit, each hairpin probe (HP) has an FA fragment at its end. In the absence of a target, the two HPs can coexist in the same system without interfering with each other. Once they are activated by a target, the two HPs will assemble into a helix, which has the two FA fragments at one side, thereby forming a complete FA that can bind the ligand fluorophore and induce fluorescence signal [20]. Although an HCR could provide a higher amplification efficiency than a target-recycled DNA circuit, it is difficult to set the FA fragments into the two HPs for HCR. On the one hand, the ends of the two HPs in HCR products are usually spaced some distance apart. On the other hand, it is easy to set the FA fragments into the two ends of one HP to induce a target-independent signal. To solve this problem, researchers designed a stem-extended HP to block the interaction between the FA fragments at the two ends of the HP [7,21]. However, the hairpin structure is metastable, and the spatial proximity further increases the probability of target-independent signal generation. Some existing FA-based hairpin self-assembly signal amplification systems are listed in Figure S1.

In this study, we attempted to find a method to construct an FA-based HCR system with a high S/B, which is essential for sensitive molecule imaging. Inspired by a remote toehold-mediated strand displacement reaction [22], we speculated that it could be possible to construct an FA-centered HCR (FAC-HCR) mode, in which the FA fragments can be spatially separated into two probes to avoid a target-independent signal, and in which no additional probe needs to be added. In addition, both four-hairpin- and two-hairpin-based HCRs were designed and compared. Apurinic/apyrimidinic endonuclease 1 (APE1), an endonuclease that plays important roles in the DNA base excision repair (BER) pathway and is involved in many pathological processes, including mutagenesis, carcinogenesis, etc. [23,24], was selected to be a model target. Moreover, the FAC-HCR system can be delivered into living cells by polyethyleneimine nanoparticles (PEI NPs), which is an established gene carrier that is made of cationic polymer PEI. PEI-NPs can efficiently adsorb electronegative nucleic acids by electrostatic adsorption and deliver the loaded nucleic acid into the cells by endocytosis [25,26,27,28,29]. Through imaging the activity of APE1, the BER process in living cells could be monitored.

## 2. Materials and Methods

### 2.1. Materials

Oligonucleotides (listed in Tables S2–S5) were synthesized from Sangon Biotechnology Co., Ltd. (Shanghai, China). APE1 was purchased from New England Biolabs (Beijing, China). DFHBI-1T, KCl, MgCl_2_, and polyethylenimine (PEI) were purchased from Aladdin (Shanghai, China). Ferric chloride hexahydrate (FeCl_3_·6H_2_O) was purchased from Macklin (Shanghai, China). Cell Counting Kit 8 (CCK-8) was purchased from Adamas-life (Shanghai, China).

Field emission scanning electron microscopy (FE-SEM) micrograph was obtained from S-4800 FE-SEM (Hitachi, Tokyo, Japan). Diameter and ζ-potential of PEI nanoparticles (PEI-NPs) were performed using Zetasizer Pro instrument (Malvern, UK). Fluorescence and absorption were measured using SpectraMax iD3 microplate reader (Molecular Devices, San Jose, CA, USA). Confocal laser scanning microscopy (CLSM) images of cells were acquired with Nikon AX with NSPARC CLSM system (Nikon, Tokyo, Japan) and FV3000 confocal microscope.

### 2.2. Design and Preparation of Probes

The structure of the HP used for 4H-HCR, 2H-HCR, and FAC-HCR was simulated and analyzed using NUPACK 4.0.1.9 online (https://www.nupack.org). All of the HPs were dissolved in RNase-free water. To enable the HP to form an ideal stem-loop structure, 10 μM of the HP was added into 1× reaction buffer (40 mM HEPES, 150 mM KCl, and 5 mM MgCl_2_) and heated to 95 °C for 5 min, followed by being slowly cooled down to room temperature (25 °C) at a rate of 2 °C/min. Then, the prepared probes were stocked at 4 °C for further use.

### 2.3. HCR

For the HCR, which includes 4H-HCR, 2H-HCR, and FAC-HCR, the corresponding HPs were mixed with 125 nM Trigger (T) in 1× reaction buffer and incubated at 37 °C for 2 h. The concentrations of the HPs are provided in the corresponding figure captions. The feasibility of the HCR was first confirmed by 10% native polyacrylamide gel electrophoresis (PAGE) and SYBR Gold staining. To further demonstrate the availability of FA, the gel was also stained by DFHBI-1T. The fluorescence signal of the DFHBI-1T-stained sample was further analyzed using a microplate reader (λ_ex_: 450 nm, scan range: 490–600 nm).

### 2.4. In Vitro APE1 Detection

For APE1 detection, the APE1 recognition probe (ARP) was first prepared according to the HP preparation process described above. Then, 125 nM ARP, 250 nM FAC-H1, and 250 nM FAC-H2 were mixed with a certain concentration of APE1 in 1× reaction buffer and incubated at 37 °C for 2 h. After that, the samples were also analyzed by PAGE and microplate reader.

### 2.5. Construction and Characterization of Carrier

PEI NPs were synthesized following previous reports [30]. Briefly, 0.2 mg of PEI (1800 Da) and 0.5406 g FeCl_3_·6H_2_O were dissolved in 100 mL deionized water and then heated to 80 °C for 4 h under magnetic stirring. After that, the PEI NPs were centrifuged and washed with ethanol three times. Finally, the PEI NPs were collected and re-suspended in deionized water for further use. To verify the delivery ability of PEI NPs, 40 μL 2 μM Cy5-labeled DNA probes (Cy5-DNA) was incubated with 100 μL 1.2 mg/mL PEI-NPs and 60 μL H_2_O at room temperature for 1 h. For APE1 imaging, the Cy5-DNA was replaced by 40 μL FAC-H1, FAC-H2, and ARP (each 2 μM). Then, the mixture was centrifuged at 12,000 rpm for 5 min to wipe off the unloaded probes and washed with 1× reaction buffer three times. The obtained mixture was stored at 4 °C for further use.

### 2.6. Cell Culture

The human astroblastoma cell lines (U-87 MG, U251) and the human embryonic kidney cell lines (HEK-293T cells) were cultured in DMEM supplemented with 10% fetal bovine serum (FBS) and 1% penicillin/streptomycin and incubated at 37 °C with 5% CO_2_. A standard CCK8 assay was used to investigate the biocompatibility of the FAC-HP-loaded PEI-NPs. Briefly, 1 × 10^4^ cells/well of the U87 MG were incubated with PEI-NPs that were loaded with different concentrations of HPs (0, 25, 50, 100, 200, and 400 μg/mL) for FAC-HCR at 37 °C for 24 h. After discarding the medium, 100 μL of serum-free medium containing CCK-8 (V_CCK8_:V_medium_ = 1:10) was added to each well and incubated for 2 h. The absorption of the samples at 450 nm was tested using a microplate reader. The CCK-8 solution and the contained cell mediums were used as the negative controls. The cell viability was calculated according to the following equation: Cell viability = (OD_450,treated_ − OD_450,blank_)/(OD_450,control_ − OD_450,blank_).

### 2.7. APE1 Imaging in Living Cells

U-87 MG (CVCL_0022, Cellosaurus), U251 (CVCL_0021, Cellosaurus), and HEK-293T (CVCL_0063, Cellosaurus) cells were plated in 15 mm glass-bottomed culture dishes (NEST Biotechnology) and cultured in DMEM for 12 h, followed by incubation with the probe-loaded PEI-NPs. Subsequently, the cells were washed with phosphate-buffered saline (PBS) and then cultured in DMEM containing 40 μM DFHBI-1T at 37 °C for 30 min. Finally, the cells were washed with PBS three times and imaged by confocal microscope (λ_ex_: 488 nm).

## 3. Results

### 3.1. Four-HP-Mediated HCR (4H-HCR)

**Figure 1:**
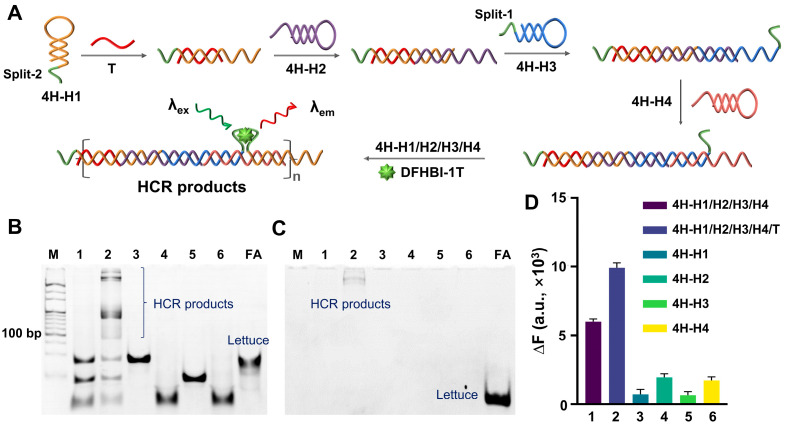
Feasibility analysis of 4H-HCR. (**A**) Principle diagram of 4H-HCR. The four HP are represented in different colors, and the green lines indicate the split FA. (**B**,**C**) PAGE results for 4H-HCR. Gel was stained by SYBR Gold and DFHBI-1T (10 μM), respectively. Lane 1: 4H-H1/H2/H3/H4. Lane 2: 4H-H1/H2/H3/H4/T. Lane 3: 4H-H1. Lane 4: 4H-H2. Lane 5: 4H-H3. Lane 6: 4H-H4. M is 20 bp DNA ladder. FA is complete single-stranded Lettuce. (**D**) Fluorescence analysis results, wherein DFHBI-1T was 2 μM. ΔF = F − F_0_, wherein F is measured fluorescence signal, and F_0_ is fluorescence signal from free DFHBI-1T. In all of these experiments, T was 2.5 μM, and all of the 4H-H1s, 4H-H2s, 4H-H3s, 4H-H4s, and FAs were 5 μM.

First, the fluorogenic ability of Lettuce or split-Lettuce and the corresponding ligand DFHBI-1T were analyzed by fluorescence spectroscopy (Figure S1). It can be seen that both Lettuce and split-Lettuce can induce an obvious fluorescence signal, while the signal from the split-Lettuce is lower. Then, we tried to construct an FA-based HCR system. First, we designed an HCR system containing four HPs (4H-H1, 4H-H2, 4H-H3, and 4H-H4), wherein the two fragments of Lettuce (split-1 and split-2) were set at the ends of 4H-H1 and 4H-H3, respectively. As in previous reports [31,32], after being activated by the trigger (T), the four HPs would form a nicked double-stranded product (Figure 1A). The PAGE results (Figure 1B) confirmed that the four hairpin probes can coexist in a system in the absence of a T without interfering with each other, while they form long products in the presence of a T. Also, the gel was stained by DFHBI-1T. The results (Figure 1C) demonstrated that only T-induced products can bind DFHBI-1T and emit a fluorescence signal. In contrast, neither single nor four HPs have visible bands. The analysis results of the fluorescence spectroscopy (Figure 1D) are consistent with those of the PAGE. Therefore, the 4HP-HCR is feasible. However, it can also be seen that the blank control without a T displayed a significant fluorescence signal, which would greatly limit the imaging performance.

### 3.2. Two-HP-Mediated HCR (2H-HCR)

**Figure 2:**
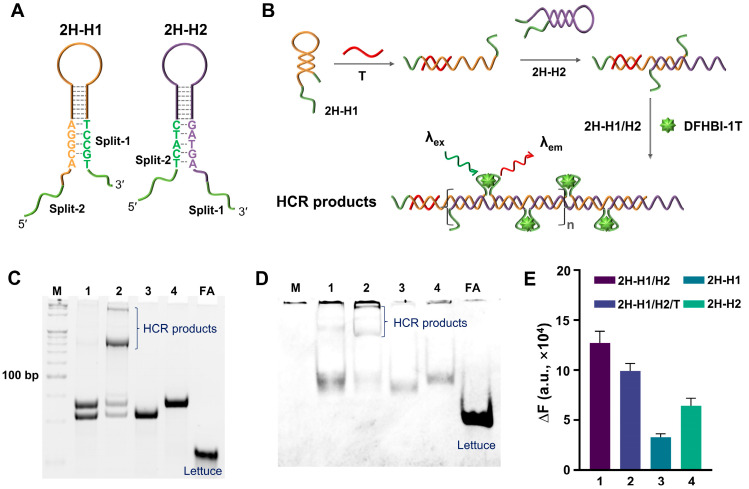
Feasibility analysis of 2H-HCR. (**A**) The structural representation of 2H-H1 and 2H-H2. The green lines indicate the split FA. (**B**) The principle diagram of 2H-HCR. Wherein the red line represents the trigger (T), and the green lines indicate the split FA. (**C**,**D**) PAGE results for 2H-HCR. The gel was stained by SYBR Gold and DFHBI-1T (10 μM), respectively. Lane 1: 2H-H1/H2. Lane 2: 2H-H1/H2/T. Lane 3: 2H-H1. Lane 4: 2H-H2. FA is the complete single-stranded Lettuce. (**E**) Fluorescence analysis results. The DFHBI-1T was 2 μM. In all of these experiments, the T was 2.5 μM, and all of the 2H-H1s, 2H-H2s, and FAs were 5 μM.

Considering that the 4H-HCR is relatively complicated and inefficient, a 2HP-HCR formed by two HPs (2H-H1 and 2H-H2) was also constructed and tested. The split-Lettuce fragments were set into the two ends of the HPs. To avoid the direct interaction of the fragments in the absence of a T, five bases of the fragments were blocked into the stem structure (Figure 2A). The second structure of the two HPs was simulated by NUPACK (Figure S2). The reaction scheme of the 2HP-HCR is shown in Figure 2B. In principle, the 2H-HCR will have a higher signal amplification efficiency, because the density of the split-Lettuce on the 2H-HCR products can be higher than that of the 4H-HCR. The feasibility of the 2H-HCR was verified by PAGE and SYBR Gold/DFHBI-1T staining (Figure 2C,D). From the results, it can be seen that although the sample with a T displayed an obvious band of HCR products, there was also a faint band produced by the control sample without a T. Worse still, the HP can be stained by DFHBI-1T directly, which means that the blocked base did not work. The fluorescence results (Figure 2E) also showed that the blank control sample without a T had a similar fluorescence intensity compared with the sample with a T.

### 3.3. FA-Centered HCR (FAC-HCR)

**Figure 3:**
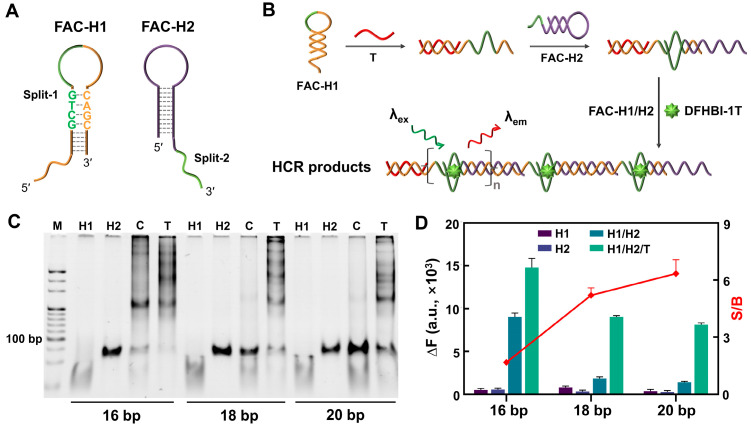
Feasibility analysis of FAC-HCR. (**A**) The structural representation of FAC-H1 and FAC-H2. The green line and letters represent the split FA. (**B**) The principle diagram of FAC-HCR. The green lines represent the split FA. (**C**) PAGE results for FAC-HCR mediated by different FAC-H1s with various stem length. The gel was stained by SYBR Gold. T is the test sample with 125 nM T. C is the control sample without a T. (**D**) Fluorescence analysis results for FAC-HCR mediated by different FAC-H1s with various stem lengths. S/B represents the ratio of the fluorescence signal of the sample containing a T to that without a T. DFHBI-1T was 2 μM. In all of these experiments, the T was 125 nM, and all of the FAC-H1s and FAC-H2s were 250 nM.

Based on the remote toehold-mediated strand displacement reaction [22], an FA-centered HCR (FAC-HCR) was designed (Figure 3A). The reaction principle is shown in Figure 3B. The split-1 of Lettuce was designed as a part of the loop of the FAC-H1, and the other fragment of Lettuce, split-2, was designed as a part of the toehold of the FAC-H2. In the absence of a T, FAC-H1 and FAC-H2 are independent of each other in one system. When the T is introduced, it can recognize and bind the toehold of FAC-H1 and then unfold the hairpin structure of FAC-H1, whose released single strand can further recognize the toehold of the FAC-H2 and unfold it. Although the complementary regions of the FAC-H1 and the FAC-H2 are separated by the split-2, the 3′-end of the FAC-H2 can also cross the spacer to hybridize with FAC-H1. Next, the released single strand of FAC-H2 can unfold another FAC-H1, and so on. Finally, the FAC-HCR products will form periodic non-complementary regions, which are the split-Lettuces for DFHBI-1T binding. Considering that the stem length of FAC-H1 is the essential effective factor for FAC-HCR, three FAC-H1s with different stem lengths were designed and tested. The PAGE result in Figure 3C show that all of the samples with a T generated obvious bands of HCR products, while the FAC-H1 with a longer stem induced less background leakage. The variations in the fluorescence signal were further analyzed by fluorescence spectroscopy. As shown in Figure 3D, the FAC-H1-20bp-mediated FAC-HCR obtained the largest signal-to-background ratio (S/B).

### 3.4. Performance of FAC-HCR

**Figure 4:**
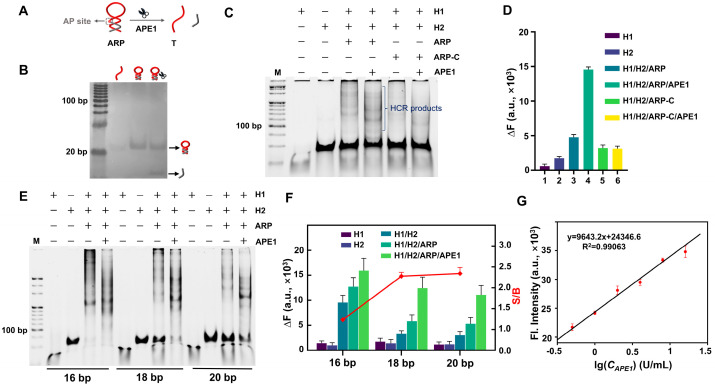
Feasibility analysis of FAC-HCR for APE1. (**A**) Principle diagram of ARP for APE1 (1.5 U/mL). (**B**) PAGE results for ARP for APE1. Gel was stained by sliver. Both T and ARP were 125 nM. (**C**) PAGE results for APE1-triggered FAC-HCR. Lane 1: H1, Lane 2: H2, Lane 3: H1/H2/ARP, Lane 4: H1/H2/ARP/APE1, Lane 5: H1/H2/ARP-C, Lane 6: H1/H2/ARP-C/APE1. Gel was stained by SYBR Gold. M is 20 bp DNA ladder. (**D**) Fluorescence analysis results. DFHBI-1T was 2 μM. ARP-Cs were 125 nM. (**E**) PAGE results for FAC-HCR for APE1. Gel was stained by SYBR Gold. (**F**) Fluorescence analysis results for FAC-HCR for APE1. (**G**) Fluorescence intensity of FAC-HCR as function of concentration APE1. Dotted line indicates limit of detection (LOD) of APE1, wherein DFHBI-1T was 2 μM. In all of these experiments, ARP was 125 nM, and both FAC-H1 and FAC-H2 were 250 nM.

APE1, an important BER-involved endonuclease was selected to serve as the model target. Unlike nucleic acids (NAs) that can directly trigger an HCR by base pairing, non-NA targets need an extra probe to convert the enzyme reaction into a trigger. Accordingly, an APE1 recognition probe (ARP, Figure 4A) in which the T is blocked by a complementary sequence that bears an apurinic/apyrimidinic (AP) site was designed. Only when the ARP is recognized and cleaved by the APE1 can the T be released and trigger an FAC-HCR. The effect of APE1 on the ARP was tested by a PAGE, and the result is shown in Figure 4B. It can be seen that the sample with APE1 displayed a low band, which was generated by the released cleavage fragment. Although both the T and ARP were 125 nM, the T generated a blurry band because the single-stranded oligonucleotides are harder to stain than double-stranded DNA. Moreover, the position of the ARP band was only slightly higher than the T band because the ARP is only 8 nt longer than the T. To avoid the shorter cleaved fragment moving out of the gel, the electrophoresis time cannot be set to long to make the difference in the migration distance between the ARP and T more significant.

Furthermore, the feasibility of the FAC-HCR for APE1 detection was also verified by a PAGE (Figure 4C) and fluorescence analysis (Figure 4D), respectively. The PAGE results showed that the sample with APE1 generated an obvious band of HCR products, while the control sample without APE1 also generated weak bands that were higher than the bands of H1 and H2, which means that the system still had a slight background leakage. In addition, the ARP-C did not generate an obvious band of HCR products, which confirms that the HCR products were specifically induced by APE1. The fluorescence analysis results are consistent with those of the PAGE. The FAC-HCRs performed with three different H1s with various stem lengths (16, 18, and 20 bp) were also analyzed by PAGE (Figure 4E) and fluorescence (Figure 4F). It can be seen that the introduction of an ARP induced some non-specific amplification, which may be because the ARP increased the amount of FAC-H1s and/or perturbation H2s, and the H1 with a longer stem caused a decreased detection signal and background signal. Among them, FAC-H1-20bp had the highest S/B. Therefore, FAC-H1-20bp was selected to perform subsequent experiments.

To analyze the sensitivity of the FAC-HCR for APE1, a series of APE1s with different concentrations (0, 0.5, 1, 2, 4, 8, and 16 U/mL) were tested by means of fluorescence spectroscopy. As expected, the intensity of the fluorescence signal increased with the increase in APE1 concentration (Figure 4G). The linear analysis (Figure 4G) demonstrated that the tested fluorescence intensity is proportional to the concentration of APE1 from 0.5 to 16 U/mL (y = 9643.2 × x + 24346.6, R^2^ = 0.99063). The detection limit was calculated to be 0.1174 U/mL (σ = 3).

### 3.5. Delivery of FAC-HCR

**Figure 5:**
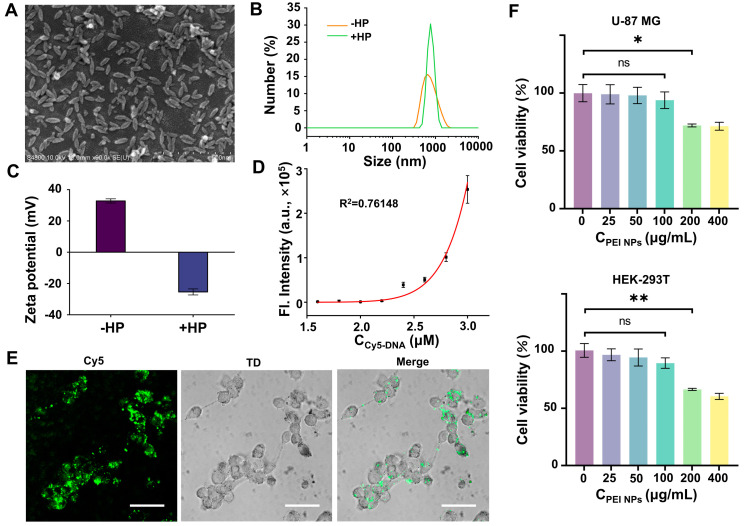
Feasibility analysis of FAC-HCR for APE1. (**A**) SEM images of PEI NPs. Scale bars, 500 nm. (**B**) Hydrodynamic size distribution of PEI NPs with or without HP. (**C**) ζ-potential of PEI NPs with or without HP. (**D**) Fluorescence detection result for supernatant, which were obtained by incubating PEI NPs with different concentrations of Cy5-DNA (1.6, 1.8, 2.0, 2.2, 2.4, 2.6, 2.8, and 3.0 μM) and centrifuging the mixture. (**E**) CLSM images of U-87 cells that were treated with Cy5-DNA-absorbed PEI NPs. TD is transmitted light channel. Scale bars, 50 μm. (**F**) Cell viability of U-87 MG cells (**top**) and HEK-293T cells (**bottom**) after being treated with indicated concentrations of FAC NPs (two-tailed Student’s *t*-test: ** *p* < 0.01, * *p* < 0.05. All bars show mean ± SEM, σ = 3).

To deliver the FAC-HCR system into living cells, PEI NPs that possess a lot of positive charges were prepared to adsorb negatively charged NA onto their surface. The NPs were first characterized by field emission scanning electron microscopy (FE-SEM), and the image (Figure 5A) shows that the NPs have uniform spindle structures with a length-to-diameter ratio of ~2.11. The average length and width were obtained by ImageJ. Figure 5B displays that the hydrodynamic size of the NPs is 599 ± 63 nm. After adsorption by FAC-H1, FAC-H2, and ARP, the hydrodynamic size of the NPs increased to 858 ± 153 nm. Moreover, the ζ-potential of the NPs was positive, while it became negative after adsorbing the HPs (Figure 5C). According to these results, it can be confirmed that the NPs can effectively adsorb the probes. Further, to quantitatively evaluate the adsorption efficiency of the NPs for NA, a cy5-labeled probe (Cy5-DNA) was incubated with the NPs at various concentrations. After centrifugation, the fluorescence of the supernatant was measured using a microplate reader. The results (Figure 5D) showed that up to 2.2 μM of Cy5-DNA can be almost completely adsorbed by 120 μg, resulting in the supernatant having no obvious fluorescence. In contrast, excess Cy5-DNA remained in the supernatant solution and emitted fluorescence, which increased with the growth of the Cy5-DNA concentration. Images from the confocal laser scanning microscope (CLSM) also confirmed that the NPs can deliver Cy5-DNA into living cells (Figure 5E). The biocompatibility of the NPs was tested by CCK8. The result indicated that the NPs cannot induce obvious cell death when their concentration does not exceed 100 μg/mL. Therefore, the concentration of the NPs used in the following cell imaging experiments was 100 μg/mL.

### 3.6. APE1 Imaging

**Figure 6:**
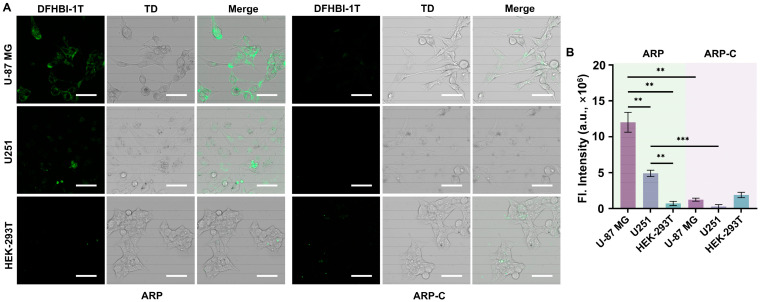
The detection ability of the FAC-HCR for APE1 in living cells. (**A**) CLSM images of the APE1 induced by U-87 MG cells, U251 cells, and HEK-293T cells. The concentrations of the ARP, ARP-C, FAC-H1, and FAC-H2 are all 2 μM. TD is the bright field. Scale bar, 50 μm. (**B**) The fluorescence analysis results for the CLSM images (two-tailed Student’s *t*-test: ** *p* < 0.01, *** *p* < 0.001. All bars show mean ± SEM, σ = 3).

The imaging ability of the FAC-HCR for APE1 in living cells was analyzed by CLSM. As shown in Figure 6A, the images demonstrated that after incubation with the PEI-NP-loaded FAC-HCR system, the U-87 MG cells that have been reported to have over-expressed APE1 [33,34,35] displayed a significant fluorescence signal. In comparison, another glioma cell line, U251, which is reported to have a lower APE1 expression level, which is responsible for the radiotherapy sensitivity [35], displayed a weak fluorescence signal. Also, the HEK-293T, a normal human cell that was used as the control [36,37], did not display an obvious fluorescence signal. To further confirm that the fluorescence signal was caused by APE1 rather than other nucleases, a control ARP probe without an AP site (ARP-C) was also delivered into the three cell lines along with the FAC-H1 and H2. The images show that the ARP-C did not cause an obvious fluorescence signal in any of the three cell lines, which verified the detection specificity of the FAC-HCR. The analysis results showed that the fluorescence signal in U-87 MG was ~2.4-fold higher than the signal of U251 and ~16.9-fold higher than the signal in HEK-293T.

## 4. Discussion

An HCR is an entropy-driven assembly reaction, usually consisting of several HPs. In comparison with enzymatic NA amplification, an HCR displays a moderate signal amplification efficiency. However, its simplicity makes it suitable for sensitive molecular imaging in living cells. For imaging, the HPs are often covalently modified by fluorochrome-fluorochrome or fluorochrome–quencher pairs. After being triggered by a target, the structural transformation of an HP induces the shortening or separation in spatial distance, thereby changing the fluorescent state of the fluorochrome. To obtain optimal fluorescence conversion efficiency, it is necessary to carefully design the location of the fluorochrome–quencher. Moreover, the intrinsic fluorescence of the fluorochrome can easily cause a background signal and is difficult to totally quench using a single quencher.

An FA is an oligonucleotide sequence without any fluorochrome, but it can become fluorescent when binding with a unique small molecule, which is also non-fluorescent in its free state. This combination-induced fluorescence mode makes it possess a negligible background signal and serves as ideal alternatives for target-responsive biosensing and imaging. Compared with the subtly designed small molecular probe that can be used to detect enzyme activity [38,39,40], the structural programmability and predictability of nucleic acids make an FA a flexible component of nucleic acid-based signal amplification systems and thus obtain a higher sensitivity. Moreover, the creation of a binary aptamer further broadens the applications of FAs. Many entropy-driven catalytic hairpin assembly systems (CHAs) have utilized FAs as reporters to achieve sensitive molecule detection [7,12,14,15]. Compared with the enzyme-mediated signal amplification system that is usually used to achieve highly sensitive detection in vitro, a CHA has a moderate amplification efficiency. However, a CHA requires few components other than HPs, such as enzymes and dNTP, and has no strict requirements for temperature and ionic strength, which makes it particularly suitable for molecular imaging of living cells [41,42,43], even in vivo [36,44], where it is difficult to add exogenous enzymes and control the reaction conditions.

Another hairpin assembly-based signal amplification system, an HCR, which has a higher amplification efficiency than a target-recycled CHA, rarely uses an FA as the reporter, because the location of the FA leads to problems. Unlike fluorochrome–quencher pairs, which can be modified at almost any site of the oligonucleotide, determining the location of the FA requires consideration of the whole reaction. A classic HCR consists of two HPs, which could form interlaced hybrids after being activated. As a result, it is difficult to make the split FA fragments close to each other on the HCR products. Therefore, some studies set the split FA fragments onto the two ends of one HP, preventing the fragments from generating target-independent interactions by blocking a part of the FA with base pairing. However, the metastability of the HP structure causes significant background leakage.

In this study, we attempted to develop an FA-based HCR with low background leakage and high sensitivity. The most feasible option is to design the FA as located at the center of the double-stranded HCR products. This means that the split FA fragments would be set at the loop of the two HPs for the HCR. This spatial separation could decrease the target-independent interaction between the split FA fragments. The experiments’ results also confirmed that the FAC-HCR has the highest S/B. The feasibility of the FAC-HCR for APE1 imaging in live cells was also verified. In conclusion, this method provides a new way to develop FA-based signal-amplified molecule imaging. To further develop this, there is still some background leakage limiting the imaging quality. We speculated that the background leakage was caused by the unstable HP structure. Many methods can improve the stability of HPs, including modifying the phosphate diester bond [45], changing the helical state of the stem [46], adding an extra mismatched base, and so on. In addition, the content of the APE1 in the nucleus and other organelles is also distinct. In principle, the FAC-HCR can be used to image APE1 in organelles, as long as the HPs can be specifically transported to the target organelle. For instance, the PEI NP can be functionalized with a nuclear localization sequence so that it can specifically transport the loaded probes on its surface into the nucleus for APE1 imaging. Of note, the PEI NP carrier used in this study is relatively large for the organelles. It is necessary to design a smart carrier, so that it can release the loaded probes after entering the cell; thereby, the HP with the targeting molecule can enter a unique organelle for the reaction. With smart design, we believe that the FA-based HCR will achieve improved performance and broader applications.

## Data Availability

Data are contained within the article and Supplementary Materials.

## Supplementary Materials

The following supporting information can be downloaded at https://www.mdpi.com/article/10.3390/bios14060274/s1: Table S1: Different designs of two HP-based self-assembly systems; Table S2: The sequences of the complete or split FAs; Table S3: The sequences of the probes for the 4H-HCR; Table S4: The sequences of the probes for the 2H-HCR; Table S5: The sequences of the probes for the FAC-HCR; Figure S1: The structure and fluorescence ability of Lettuce and split-Lettuce; Figure S2: Secondary structures and Gibbs free energy change of 2H-H1 and 2H-H2, simulated by NUPACK.
