# Supplementary material for: Fluorogenic Aptamer-Based Hybridization Chain Reaction for Signal-Amplified Imaging of Apurinic/Apyrimidinic Endonuclease 1 in Living Cells
Source: Biosensors (Basel). 2024 May 27;14(6):274. doi: 10.3390/bios14060274 (PMC11202136; doi:10.3390/bios14060274)
Supplement: Supplementary file 1 [file biosensors-14-00274-s001.zip › biosensors-2979053-supplementary.pdf]

biosensors

MDPI

Supplementary Material

Fluorogenic Aptamer-Based Hybridization Chain Reaction for Signal-Amplified Imaging of Apurinic/Apyrimidinic Endonuclease 1 in Living Cells

Meixi Liu [1,2,†], Yunjie Tan [1,2,†], Chen Zhou [1,2,†], Zhaoming Fu [1,2], Ru Huang [1,2,*], Jin Li [3,*] and Le Li [4]

[1] State Key Laboratory of Digital Medical Engineering, School of Biomedical Engineering, Hainan University, Sanya 572024, China
[2] Key Laboratory of Biomedical Engineering of Hainan Province, One Health Institute, Hainan University, Sanya 572024, China
[3] Department of Painology, Hainan Cancer Hospital, Haikou 570311, China
[4] NHC Key Laboratory of Tropical Disease Control, Hainan Medical University, Haikou 571199, China
* Correspondence: huangru@hainanu.edu.cn (R.H.); hnszlyyttk@163.com (J.L.)
† The authors contribute equally in this study.

Table S1. Different design of two HP based self-assembly systems[§].

Methods	FA	Probes' Design	Features	Ref.
CHARGE	Broccoli	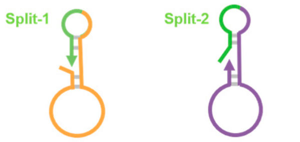	The split FA fragments are set at the end of the two HP and are blocked by the extended stem respectively.	[1]
INSIGHT	Broccoli	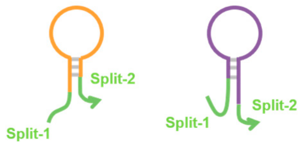	The split FA fragments are set at the two ends of the two HP and one of the split is blocked by the extended stem.	[2]
EXO-HCR	Aptamer for NMM	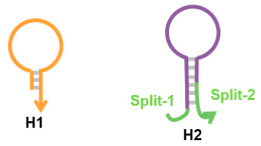	The split FA fragments are set at the two ends of one HP and one of the split is blocked by the extended stem.	[3]
FAC-HCR	Lettuce	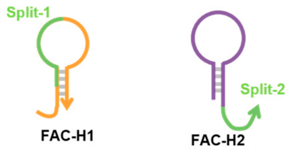	One of the split is set at the loop of a HP, and the other split is set as a toehold of the other HP.	Our work

§ CHARGE: Catalytic Hairpin Assembly RNA circuit that is Genetically Encoded. INSIGHT: IN SItu Genetic Hybridization amplification Technique. EXO-HCR: Exonuclease III-assisted DNA cycling and hybridization chain reaction. NMM: N-nethyl mesoporphyrin IX.

Table S2. The sequences of the complete or split FA.

Name	Sequence(5′→3′)
**Lettuce**	GTCTTAGT AGGGATGATGCGGCAGTGGGCT TCATCGAA CAGTGTTTA TTCGATGA GGGG ACTAAGAC
**Substrate**	CCAGGAACTAATCAGACAAG G AACTGATTACAAACA
**Split-1**	TAGTAGGGATGATGCGGCAGTGGGCT TCATC CTTGTCTGATTAGTTCCTGG
**Split-2**	TGTTTGTAATCAGTT TGATGA GGGG ACTA

Note: The underlines indicate the complementary base among the oligonucleotides.

Table S3. The sequences of the probes for 4H-HCR.

Name	Sequence(5′→3′)
**Trigger**	GAGCTTCATCTTCATCTCCGAGACTTC
**4H-H1**	TAGTAGGGATGATGCGGCAGTGGGCTTCATCGAAGTCT CGGAGATGAAGATGAAGC CATCGT GCTTCATCTTCATCT-CCG
**4H-H2**	GCTTCATCTTCATCTCCGGTTTTGCGGAGATGAAGATGAAGCACGATG
**4H-H3**	CAAAAC CGGAGATGAAGATGAAGC TTGCCT GCTTCATCTTCATCTCCGTGATGAGGGGACTA
**4H-H4**	GCTTCATCTTCATCTCCG AGACTTC CGGAGATGAAGATGAAGC AGGCAA

Note: The underlines indicate the intramolecular complementary base of the probes. The green fonts represent the split Lettuce sequences.

Table S4. The sequences of the probes for 2H-HCR.

Name	Sequence(5′→3′)
**2H-H1**	TAGTAGGGATGATGCGGCAGTGGGCTTCGGAGAAGTCT CGGAGATGAAGATGAA ACTCGATCTCATC TTCATCTTC ATCTCCG AGGGGACTATTTT
**2H-H2**	TAGTAGGGATGATGCGGCAGTGGGCTTCATCTTCATCTTCATC TCCGAGACTTC GATGAAGATGAAGATGA GATCGAG-TAGATGAGGGGACTATTTT

Note: The underlines indicate the intramolecular complementary base of the probes. The green fonts represent the split Lettuce sequences.

Table S5. The sequences of the probes for FAC-HCR.

Name	Sequence(5′→3′)
**FAC-H1(16)**	GAAGTCT CGGAGATGAAGATGAA TTCTGACAGGGGACACTTAACG TTCATCTTCATCTCCG
**FAC-H2(16)**	TTCATCTTCATCTCCG AGACTTC CGGAGATGAAGATGAA CGTTAAGTGT AGGGATGATGCGGCAGTGGGCTT-GTCAGAA
**FAC-H1(18)**	GAAGTCT CGGAGATGAAGATGAA GCCTGACAGGGGACACTTCA GCTTCATCTTCATCTCCG
**FAC-H2(18)**	TTCATCTTCATCTCCG AGACTTC CGGAGATGAAGATGAA GCTGAAGTGT AGGGATGATGCGGCAGTGGGCTT-GTCAGGC
**FAC-H1(20)**	GAAGTCT CGGAGATGAAGATGAA GCTGGACAGGGGACACTT CAGCTTCATCTTCATCTCCG
**FAC-H2(20)**	TTCATCTTCATCTCCG AGACTTC CGGAGATGAAGATGAA GCTGAAGTGT AGGGATGATGCGGCAGTGGGCTT-GTCCAGC
**ARP**	GAAGTCTC/idSp/GAG CTTCATCTTCAT CTCCGAGACTTC
**ARP-C**	GAAGTCTCGGAG CTTCATCTTCAT CTCCGAGACTTC
**Cy5-DNA**	Cy5-TATAGTACCTTCGACAACGAGCCCTTGTCGAAGGT

Note: The underlines indicate the intramolecular complementary base of the probes. The green fonts represent the split Lettuce sequences. idSp represents an apurinic/apyrimidinic (AP) site. The 5′-end of the probe was functionalized with a cyanine-5 dye, represented by Cy5.

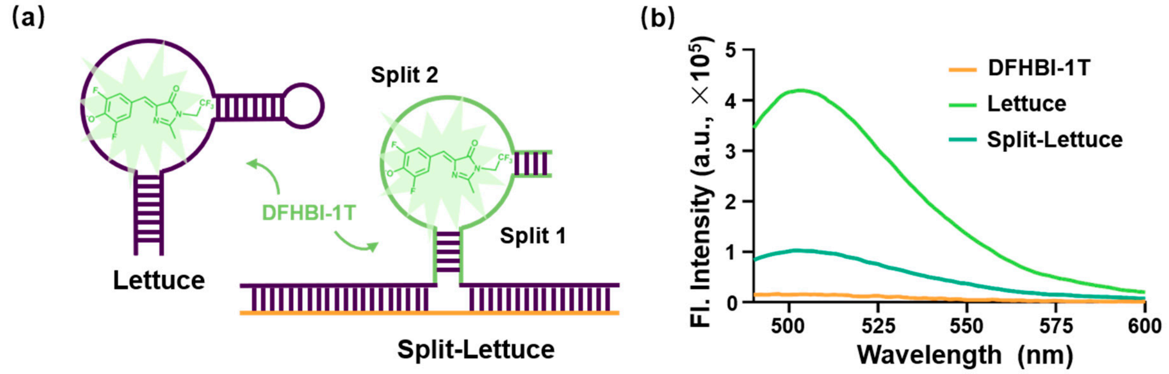

Figure S1. The structure and fluorescence ability of Lettuce and split-Lettuce. (a) The structure of Lettuce and the split-Lettuce. Lettuce can directly bind with DFHBI-1T and emit fluorescence. Split-Lettuce is formed by two DNA fragments, split-1 and split-2. Only when the split-1 and split-2 bind a single substrate simultaneously, DFHBI-1T can be recognized and emit fluorescence. (b) Fluorescence detection results of free DFHBI-1T, DFHBI-1T + Lettuce, and DFHBI-1T + split-Lettuce + Substrate. Wherein the DFHBI-1T was 2 μM, all of the Lettuce, the split-L, the split-R, the substrate were 5 μM.

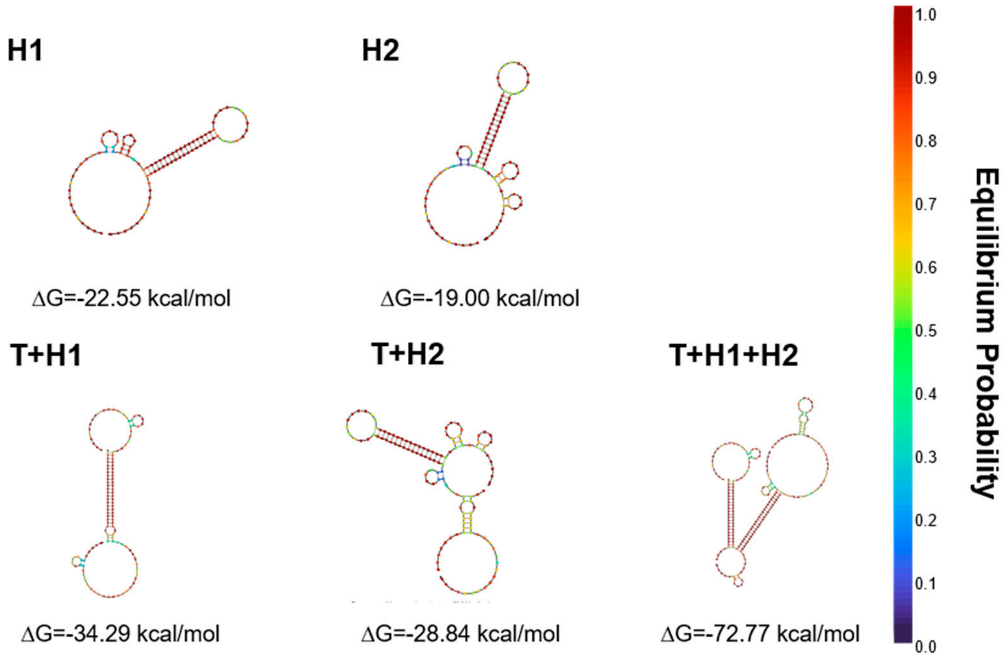

Figure S2. Secondary structures and Gibbs free energy change of 2H-H1 & 2H-H2 simulated by NUPACK.

References

1. Karunanayake Mudiyanselage, A.P.K.K.; Yu, Q.; Leon-Duque, M.A.; Zhao, B.; Wu, R.; You, M. Genetically encoded catalytic hairpin assembly for sensitive RNA imaging in live cells. *J Am Chem Soc* **2018**, 140, 8739-8745.
2. Ren, K.; Wu, R.; Karunanayake Mudiyanselage, A.P.K.K.; Yu, Q.; Zhao, B.; Xie, Y.; Bagheri, Y.; Tian, Q.; You, M. In Situ genetically cascaded amplification for imaging RNA subcellular locations. *J Am Chem Soc* **2020**, 142, 2968-2974.
3. Sun, J.; Jiang, W.; Zhu, J.; Li, W.; Wang, L. Label-free fluorescence dual-amplified detection of adenosine based on exonuclease III-assisted DNA cycling and hybridization chain reaction. *Biosens Bioelectron* **2015**, 70, 15-20.
